# Sleep quality and risk of oropharyngeal dysphagia in older people in intensive care units

**DOI:** 10.1590/2317-1782/e20250183en

**Published:** 2026-06-15

**Authors:** Isabela Cristina Monteiro de Lira, Natália de Castro e Silva Martins, Luciano Pedrosa de Oliveira, Bianca Emmanuely Clemente da Silva, Luciana Moraes Studart-Pereira

**Affiliations:** 1 Departamento de Fonoaudiologia, Universidade Federal de Pernambuco – UFPE - Recife (PE), Brasil.; 2 Instituto de Medicina Integral Professor Fernando Figueira – IMIP - Recife (PE), Brasil.

**Keywords:** Deglutition Disorders, Intensive Care Units, Sleepiness, Sleep Quality, Aged

## Abstract

**Purpose:**

To associate the risk of oropharyngeal dysphagia with poor sleep quality in older adults in intensive care units.

**Methods:**

Analytical, observational, cross-sectional study carried out in intensive care units with purposive sampling. A screening form was completed daily for patients admitted during the research period, totaling 221 participants. The inclusion criteria were older individuals (> 60 years old) with preserved cognitive status, communication skills, and physical and emotional stability; based on these criteria, the sample comprised 66 participants. The study used the Richards-Campbell Sleep Questionnaire to assess sleep quality, item 4 of the Sleep in ICU Questionnaire to assess daytime sleepiness, and the Oropharyngeal Dysphagia Screening in Hospitalized Older Adults to assess the risk of dysphagia.

**Results:**

There was a predominance of females (65.2%) and of the age group of 60-70 years (66.7%). Of these, 30.3% were hospitalized due to respiratory problems. Most of the sample (62.1%) reported good sleep quality, while 37.9% had poor sleep. Sleep depth and time to sleep lowest scores, reflecting increased sleep latency and reduced sleep depth. Also, 16.7% scored at risk for oropharyngeal dysphagia during hospitalization, with choking (24.2%) and respiratory incoordination (22.7%) being the most prevalent risk signs in the study population. The risk of oropharyngeal dysphagia was not significantly associated with poor sleep quality (p > 0.05).

**Conclusion:**

Sleep quality was not associated with the risk of dysphagia in older adults admitted to intensive care units.

## INTRODUCTION

Sleep is a fundamental pillar for preserving people's overall health^(1)^. It is one of the most essential biological functions for human beings, playing a significant role in cognitive processes. Being directly related to quality of life, sleep is currently one of the most relevant topics on the rise, as there is consistent evidence that interference in its quality can negatively influence metabolic and inflammatory processes with broad harmful impacts on human health^(1,2)^.

However, several factors can affect the distribution of sleep phases during the night, such as age, circadian rhythm, room temperature, medications, and specific sleep disorders. In addition, studies indicate that a large part of the population has complaints regarding sleep and that the prevalence of these disorders is high^(1,3)^. There is a high prevalence of sleep disorders, especially insomnia, among older people. These disorders may be related not only to aging but also to chronic non-communicable diseases^(4)^. Research indicates that another population with a high prevalence of sleep disorders is that of intensive care patients, which may be caused by factors related to the routine of care within the intensive care unit (ICU), factors intrinsic to the patients, and their acute condition^(5)^.

Given the high prevalence of sleep disorders in these settings and their impact on health, early diagnosis and appropriate treatment of these conditions are necessary. Polysomnography is considered the gold standard test for the objective identification of sleep disorders^(6)^. However, there are low-cost, sensitive, quick, and practical instruments – e.g., the Richards-Campbell Sleep Questionnaire (RCSQ) – that can be applied at the bedside as a screening tool for sleep disorders in ICUs^(7)^. The RCSQ was recently cross-culturally adapted to Brazilian Portuguese, demonstrating good interrater reliability. Its results have already been validated against polysomnography, showing excellent internal consistency and moderate correlation^(8)^.

Sleep disorders generally cause poor sleep quality and/or sleep deficit, which can lead to sleep deprivation and excessive daytime sleepiness, with negative consequences such as behavioral and physiological changes, possibly compromising human cognition and neurophysiological functions^(3)^. When this situation becomes chronic, it can increase the risk of developing neurodegenerative diseases, such as Alzheimer's disease and other types of dementia^(9)^. Moreover, sleep-related breathing disorders, such as obstructive sleep apnea (OSA), are associated with oropharyngeal dysphagia. The vibratory trauma caused by OSA and snoring may impair swallowing function. It is estimated that approximately 15% of patients with OSA exhibit symptoms of dysphagia^(10)^. There are also sleep disorders that are correlated with dysphagia in ICUs, mainly due to the association with excessive daytime sleepiness, increasing the risk of swallowing disorders and inadequate nutrition^(11,12)^. A study demonstrates an association between sleep quality and oropharyngeal dysphagia (OD) in community-dwelling older adults^(13)^. However, there is a gap in the literature on these variables in older people in the context of ICU hospitalization.

In addition to comorbidities already associated with older adults (e.g., cardiovascular diseases and dementia syndromes), aging causes physiological changes such as loss of muscle mass, weakness, sensory changes, and reduced salivary flow, which can favor the onset of dysphagia^(14,15)^. OD is a multifactorial disorder in swallowing function, with serious complications and impacts on the patient’s quality of life, such as dehydration, malnutrition, aspiration pneumonia, and increased mortality^(16)^. Thus, OD is also considered a geriatric syndrome, with a high prevalence in older adults compared to other age groups, affecting approximately 10% to 30% of this population^(14)^. In hospitalized patients, OD has a prevalence between 3% and 62%, according to the characteristics of the population studied, with the highest values found in older people^(17)^.

OD is constantly underdiagnosed in hospitals, mainly due to the lack of pre-established screening/identification protocols upon patient admission or the lack of permanent speech-language pathologists in the services. It is greatly important to use instruments in this context to facilitate the identification of this disorder and reduce morbidity, mortality, and health costs associated with the sequelae of OD^(17,18)^. The Oropharyngeal Dysphagia Screening in Hospitalized Older Adults (RADI-H, in Portuguese) is one of the validated tools used in hospitals, aiming at early diagnosis and intervention. If its score indicates the risk for OD, speech-language pathologists evaluation is recommended^(19)^.

This study aimed to verify the association between the risk of OD and poor sleep quality in older patients admitted to ICUs. It considered that verifying sleep quality and the risk of OD will provide greater knowledge about this correlation, favoring the implementation of educational, preventive, and promotional measures for good sleep quality and safe swallowing in ICUs.

## METHODS

This study complied with resolution 466/12 of the Brazilian National Health Council. The study protocol was approved by the Research Ethics Committee of the Professor Fernando Figueira Institute of Integrative Medicine (IMIP), under evaluation report number 6.884.271 and CAAE: 79645924.4.0000.5201.

This is an analytical, observational, cross-sectional study, with purposive sampling, developed from July to September 2024 in three ICUs of a referral hospital, with 10 beds each, whose patients were admitted due to clinical or respiratory changes.

The first stage of data collection was the daily screening of patients recently admitted to the ICU. It used an instrument developed by the researchers in Google Forms, with sociodemographic data and exclusion criteria; the information was collected from the patients' electronic medical records. At the end of the study period, 221 patients had been admitted to the ICUs.

**Figure 1:**
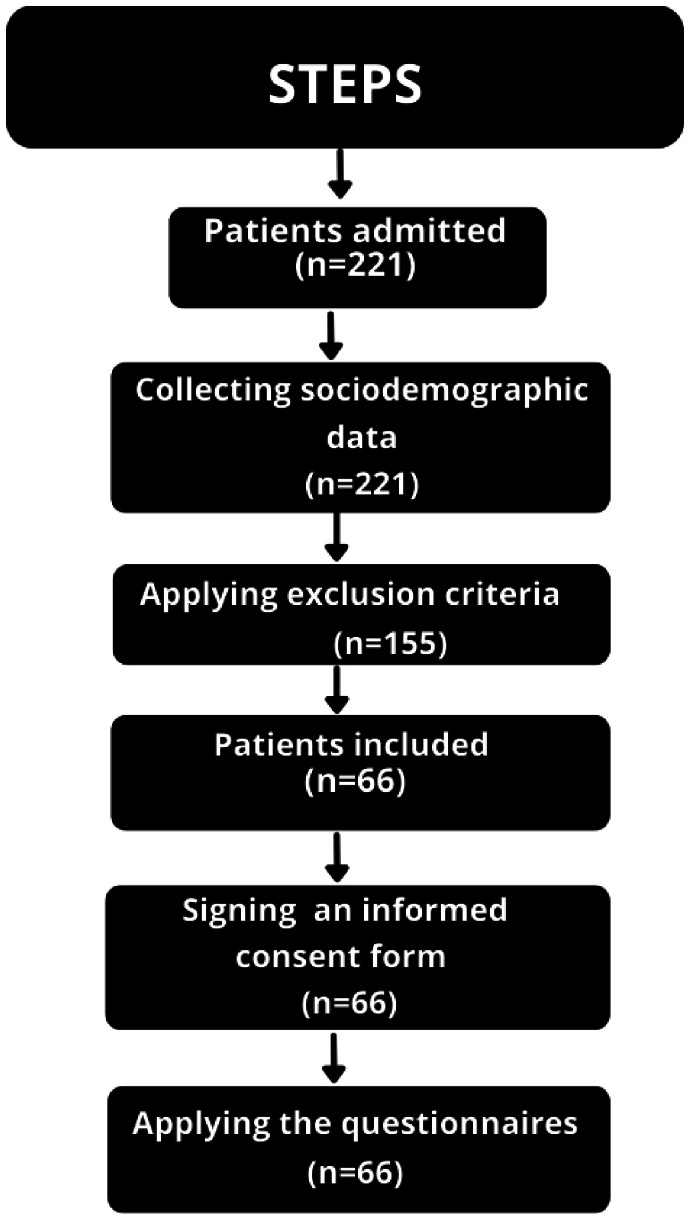
Flowchart with the research process

Subjects aged > 60 years, with preserved cognitive status, able to communicate, and with physical and emotional stability (assessed by the medical team based on clinical impressions in medical records) were eligible to participate in the study. Participants were asked to complete the questionnaires independently. Patients with amaurosis, Glasgow Coma Scale < 13, who were using sedative drugs, non-oral feeding, and patients who had undergone orotracheal intubation during their current hospitalization were excluded. The 66 eligible patients were invited to participate in the study and received an explanation of the study content and objectives. Afterwards, they were asked to read and sign an informed consent form (Figure 1).

The RCSQ^(8)^ was administered at the bedside after at least 24 hours of hospitalization. This instrument assesses the quality of sleep in patients admitted to the ICU and is the most valid and reliable tool for use in this context. It is a self-administered questionnaire with five items that assess sleep depth and latency, number of awakenings, and sleep efficiency and quality. Adapted from other studies, a sixth item was added to the questionnaire to assess nighttime noise^(8)^.

**Figure 2:**
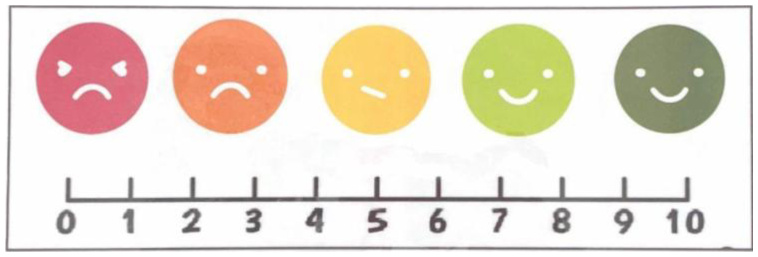
Visual Analog Scale (VAS)

Each RCSQ domain has its response recorded on a visual analog scale (VAS) from 0 to 10 cm; higher scores represent better sleep (Figure 2). The scale was presented to the participant with the following command: “On a scale of 0 to 10, your sleep last night was...”. The patient was asked to indicate the score for each category. The VAS was used from 0 cm to 10 cm to facilitate its application at the bedside. However, for RCSQ calculation purposes, it was converted to millimeters by multiplying the responses by 10. The total score is calculated from the average of the five items' scores, representing the overall perception of sleep. The sixth item was assessed individually, as instructed in the questionnaire. Patients whose total score was ≥ 50 were defined as having good sleep, and those with a total score < 50 were defined as having poor sleep^(20)^.

Item 4 of the Sleep in the Intensive Care Unit Questionnaire (SICUQ)^(21)^, which also has a cross-cultural adaptation to Brazilian Portuguese, assesses the overall level of daytime sleepiness during the ICU stay. It was also applied, using a scale from 1 to 10 (1 = unable to stay awake; 10 = fully awake), because the RCSQ does not assess this domain^(8)^.

Then, the researchers applied the RADI-H^(19)^. It has nine questions about swallowing function and its possible changes and/or suggestive signs. The answers are grouped into: “sometimes” (1 point), “always” (2 points), “yes” (2 points), “no” (0 points), and “I don’t know” (2 points), according to the frequency or absence of the symptom.

The instrument’s total score can be up to 18 points, with scores equal to or greater than 4 being considered as positive RADI-H (presence of risk for OD). Patients who scored ≥ 4 on the RADI-H were referred to the Speech-Language Pathologists Service of the hospital inpatient unit for a complete evaluation.

The evaluators mediated the responses to the questionnaire to ensure patient biosafety, using a printed and laminated VAS. Thus, the material was sanitized, reducing the risk of cross-infection. In addition, both assessments were applied by the same evaluator, who is a speech-language pathologist, with expertise in the instruments used. All the patients’ scores were generated online via Google Forms with a smartphone.

After applying the questionnaires, participants received guidance on preventive measures for bronchoaspiration to promote safe swallowing. They were also informed about the main effects of sleep deprivation on quality of life and health through general guidance on sleep hygiene and prevention of sleep disorders.

The collected data were analyzed using SPSS 26.0 (Statistical Package for the Social Sciences) for Windows and Excel 365. The results are presented in tables with their respective absolute and relative frequencies. Quantitative variables were represented by measures of central tendency and dispersion. Associations were verified using the chi-square and Fisher's exact tests for qualitative variables and the Shapiro-Wilk normality test for quantitative variables. The Mann-Whitney test was used to compare two groups for independent variables, and the Kruskal-Wallis test was used to compare more than two groups. All tests were applied with a significance level of 5% (p-value ≤ 0.05).

## RESULTS

The demographic characteristics and reasons for hospitalization of the patients included in the study are presented in Table 1. Most patients were in the age group of 60 to 70 years (66.7%), followed by those between 71 and 80 years (24.2%). Females were predominant, representing 65.2% of the sample. The reasons for hospitalization were diverse, with isolated respiratory changes being the main reason, accounting for 30.4% of the patients. Other relevant conditions included isolated oncological causes (15.3%) and the combination of respiratory changes with cardiovascular changes (10.6%).

**Table 1:** Profile of older people admitted to intensive care units

**Variables**	**n**	**%**
**Age (years)**		
60 – 70	44	66.7
71 – 80	16	24.2
81 – 90	6	9.1
**Sex**		
Males	23	34.8
Females	43	65.2
**Reason for admission**		
Respiratory changes	20	30.4
Oncological	10	15.3
Respiratory changes and cardiovascular changes	7	10.6
Clinical Multimorbidity^*^	29	43.7

(*) Cases involving the coexistence of two or more relevant clinical conditions, as respiratory and oncological changes; renal/hepatic changes and sepsis; cardiovascular changes, drug intoxication and CT; respiratory changes and renal/hepatic changes; respiratory changes and grade III obesity; cardiovascular changes; renal/hepatic changes; cardiovascular and oncological changes; gastrointestinal changes, renal and oncological changes; sepsis; respiratory, renal and oncological changes; renal changes, sepsis and oncological changes; gastrointestinal and oncological changes; cardiovascular changes and gastrointestinal changes; oncological + postoperative.

The score mean of sleep depth was 43.8 ± 37.2, with a median of 50.0 and values ranging from 0.0 to 100.0. The time needed to sleep had a mean of 47.9 ± 33.4, a median of 50.0, and a range from 0.0 to 100.0. These two parameters had the lowest scores, reflecting increased sleep latency and reduced sleep depth. Noise, as an influencing factor, was reported with a mean of 71.1 ± 32.0, a median of 80.0, and a range from 0.0 to 100.0, being considered as little nighttime noise. Regarding the overall level of daytime sleepiness, participants reported a score mean of 6.2 ± 2.7, with a median of 6.0 and values ​​ranging from 1.0 to 10.0, indicating the presence of daytime sleepiness, since a score of 10 would reflect the absence of sleepiness (Table 2).

**Table 2:** Variables related to sleep quality (RCSQ) and daytime sleepiness (SICUQ) in older patients during their stay in intensive care units

**RCSQ items** ^*^	**Mean ± SD**	**Median (Q_1_; Q_3_)**	**Minimum – Maximum**
Sleep depth	43.8 ± 37.2	50.0 (0.0; 80.0)	0.0 – 100.0
Time to sleep	47.9 ± 33.4	50.0 (20.0; 72.5)	0.0 – 100.0
Awakenings	54.4 ± 31.3	60.0 (30.0; 80.0)	0.0 – 100.0
Return to sleep	53.8 ± 35.2	60.0 (20.0; 82.5)	0.0 – 100.0
Sleep quality	55.6 ± 35.6	60.0 (20.0; 80.0)	0.0 – 100.0
Noise	71.1 ± 32.0	80.0 (60.0; 100.0)	0.0 – 100.0
Please rate your overall level of daytime sleepiness during your ICU stay (SICUQ).	6.2 ± 2.7	6.0 (4.0; 8.0)	1.0 – 10.0

(*) RCSQ - Richards-Campbell Sleep Questionnaire. SICUQ - Sleep in the Intensive Care Unit Questionnaire

All values presented represent scores assigned to each domain.

The median levels of sleepiness were similar between age groups, with no significant difference (p = 0.711). Women had values ​​close to those of men, with no statistical significance (p = 0.164). On the other hand, sleep quality was significantly different (p = 0.039), with higher levels of sleepiness among patients with poor sleep [5.0 (3.0; 8.0)] than with good sleep [7.0 (5.0; 9.0)]. The analysis of the risk of OD revealed no significant difference, with a median of 5.0 (4.0; 9.0) for patients at risk and 6.0 (5.0; 8.0) for those not at risk (p = 0.584) (Table 3).

**Table 3:** Relationship between overall levels of daytime sleepiness and age, sex, sleep quality and the risk of oropharyngeal dysphagia during the stay of older adults admitted to intensive care units

**Variables**	**Please rate your overall level of daytime sleepiness during your ICU stay**	**p-value**
	**Median (Q_1_; Q_3_)**	
**Age (years)**		
60 – 70	6.0 (4.3; 8.0)	0.711 ^*^
71 – 80	6.0 (4.0; 10.0)	
81 – 90	5.0 (4.5; 8.5)	
**Sex**		
Males	5.0 (4.0; 7.0)	0.164 ^**^
Females	7.0 (5.0; 9.0)	
**RCSQ**		
Poor	5.0 (3.0; 8.0)	0.039 **
Good	7.0 (5.0; 9.0)	
**RADI-H**		
At risk	5.0 (4.0; 9.0)	0.584 **
No risk	6.0 (5.0; 8.0)	

(*) Kruskal-Wallis;

(**) Mann-Whitney

Most of the sample (62.1%) reported good sleep quality, while 37.9% (n = 25) had poor sleep quality (RCSQ < 50). Also, 16.7% (n = 11) scored at risk of OD during hospitalization. Among patients at risk of OD, 36.4% (n = 4) had poor sleep, while 63.6% (n = 7) had good sleep. Thus, there was no significant association between the groups (p = 1.000). These findings suggest that the risk of OD did not significantly impact sleep quality or vice versa (Table 4).

**Table 4:** Association between the risk of oropharyngeal dysphagia and sleep quality in older patients admitted to intensive care units

**Variables**	**RCSQ**		**p-value**
	**Poor**	**Good**	
	**n (%)**	**n (%)**	
**RADI-H**			
At risk	4 (36.4)	7 (63.6)	1.000 ^*^
No risk	21 (38.2)	34 (61.8)	

(*) Fisher’s exact test

Regarding the risk of dysphagia, most individuals did not report significant difficulties in the swallowing process, although a few complaints were noted. Among these, the most commonly reported was choking after swallowing, with 24.2% stating it occurs sometimes, and 1.5% reporting it occurs always. Symptoms related to physical effort, such as fatigue or shortness of breath after eating, also stand out, which were reported by 22.7% of individuals as occasional occurrences and by 3.0% as constant. Moreover, 13.6% stated they sometimes needed to repeatedly swallow food for it to go down, and 12.1% reported this always occurred. Regarding the presence of throat clearing after swallowing, 12.1% indicated that it occurs occasionally, and 10.6%, frequently. Also, 10.6% indicated occasional effort to swallow, and 10.6% faced constant effort. The development of pneumonia following choking was exceptionally rare, with 98.5% not experiencing this complication, and only 1.5% reporting uncertainty about it.

Regarding age, the distribution between the groups of 60 to 70 years, 71 to 80 years, and 81 to 90 years was not statistically significantly different for either RCSQ (p = 0.725) or RADI-H (p = 0.453). In RCSQ, individuals aged 60 to 70 years had 61.4% of good sleep, followed by the groups of 71 to 80 years (68.7%), and 81 to 90 years (50.0%). In RADI-H, the proportion of risk of dysphagia was lower in all age groups, with 15.9%, 12.5%, ​​and 33.3%, respectively. Sex was not statistically significantly associated with RCSQ (p = 0.705) or RADI-H (p = 0.495) (Table 5).

**Table 5:** Association between age, sex, and sleep quality and the risk of oropharyngeal dysphagia in older patients admitted to intensive care units

**Variables**	**RCSQ**		**p-value**	**RADI-H**		**p-value**
	**Poor**	**Good**		**At risk**	**No risk**	
	**n (%)**	**n (%)**		**n (%)**	**n (%)**	
**Age (years)**						
60 – 70	17 (38.6)	27 (61.4)	0.725 ^*^	7 (15.9)	37 (84.1)	0.453 *
71 – 80	5 (31.3)	11 (68.7)		2 (12.5)	14 (87.5)	
81 – 90	3 (50.0)	3 (50.0)		2 (33.3)	4 (66.7)	
**Sex**						
Males	8 (34.8)	15 (65.2)	0.705 ^**^	5 (21.7)	18 (78.3)	0.495 *
Females	17 (39.5)	26 (60.5)		6 (14.0)	37 (86.0)	

(*) Fisher’ exact test;

(**) Chi-square test

## DISCUSSION

In this study, there was no significant difference between the risk of OD and poor sleep quality in hospitalized older patients. This differs from a published study, which identified that the risk of dysphagia is associated with sleep quality in community-dwelling older people in Japan, claiming that individuals in this age group may not perceive insufficient sleep due to increased daytime naps, but dysphagia symptoms may be more noticeable. The authors also state that the risk of dysphagia can influence all three primary dimensions of sleep (sleep duration, reduced sleep satisfaction, and low sleep efficiency), thus leading to the manifestation of unhealthy and insufficient sleep. Moreover, they emphasize the importance of considering the risk of dysphagia and sleep health as interconnected factors since there is a potential for a vicious cycle of decline in physical function due to swallowing disorders and subsequent decline in sleep quality^(13)^. This divergence in findings between studies can be justified by the distinction between the socioeconomic levels of the populations. In addition to the difference between the scenarios studied, patients often choose not to express their complaints and dissatisfaction regarding care during hospitalization.

ICU-acquired OD is multifactorial and can be due to trauma to the oral cavity, pharynx, and/or larynx caused by the endotracheal tube, neuromuscular weakness, sensory impairment, cognitive impairment or change after extubation, and incoordination between breathing and swallowing^(17)^. Dysphagia can affect even healthy older adults, as aging promotes morphophysiological changes in the swallowing mechanism, such as reduced amplitude and speed of movements, decreased muscle mass with consequent loss of strength in the speech and swallowing organs, and impairment in the pharyngeal region, such as reduced opening of the pharyngoesophageal sphincter. These changes increase the risk of food penetration and aspiration in this population^(14)^.

In this study, 30.3% of participants were hospitalized due to respiratory changes, and the patients who scored at risk for OD (16.7%) were hospitalized in one of the two respiratory ICUs. This finding corroborates several published studies, which show that the processes of breathing and swallowing occur through similar pharyngeal anatomical structures, are controlled by the brainstem, and cannot occur simultaneously^(22,23)^. Both provide important functions related to airway protection, such as a strong and effective cough, since reduced levels of cough flow are associated with a higher risk of aspiration^(24)^.

Another study^(16)^ with older adults highlighted coughing as the most common complaint during dysphagia screening. In this study, choking was the item that presented the highest occasional occurrence. Although it is a sign and symptom of dysphagia, it is often neglected. However, its presence increases the risk of aspiration pneumonia and airway compromise, more serious complications of dysphagia that can lead to death. Despite being less frequent, choking is the fourth leading cause of unintentional death due to the risk of asphyxiation, and its mortality rate in older people is higher than in any other age group^(16,17,25)^.

This study found that approximately 40% of older adults admitted to ICUs had poor sleep quality during their stay in the unit, and that lighter sleep and longer sleep latency stood out among the parameters evaluated. The literature indicates that poor sleep can lead to unfavorable outcomes and affect the patients’ recovery^(5)^. Moreover, increased sleep latency and the severity of insomnia in older patients can expose this group to the risk of death^(26)^.

However, most individuals interviewed in this study stated that they had a good night's sleep during their hospitalization. The low level of education that many users of the public health system still have may be a justification for this finding, since they are often unable to express their dissatisfaction with the quality of the service and its environmental conditions, and such issues could influence the quality of sleep^(27)^.

Furthermore, the participants did not indicate that environmental noise was a disruptive factor for their sleep during their stay. However, the literature shows that environmental noise and the checking of vital signs are the most significant contributors to sleep interruption during hospitalization^(28)^. Noise produced by health professionals continues to be the most critical factor in inhibiting sleep in wards and ICUs^(29,30)^.

Therefore, sleep promotion measures must be implemented in ICUs, despite being challenging and involving the participation of a multidisciplinary team. Research has shown that some changes in service routines (e.g., synchronizing care by implementing multiple tasks, such as administering analgesics and checking vital signs in the same visit) can provide better sleep quality indices for these patients^(5,29)^. In addition, non-pharmacological measures that are low-cost and easy to implement, such as environmental modification with a noise and light reduction program, which may involve the use of eye masks and earplugs, are also an effective way to promote sleep^(5,29)^.

In our study, patients with poorer sleep quality had greater daytime sleepiness. A study found that daytime sleepiness can increase the risk of dysphagia by up to two times^(12)^. Other studies^(26,31)^ also show that daytime sleepiness is a symptom that can be related to psychiatric and neurological disorders and to pulmonary and cardiac changes. It is also frequently associated with sleep disorders, such as obstructive sleep apnea and insufficient sleep^(31)^.

Lastly, as a limitation of the study, we highlight the exclusive use of self-reported instruments for data collection. Although the applied questionnaires are validated and widely used in the literature, the data should be interpreted with caution, given the inherent subjectivity of self-perception.

Furthermore, the sample size resulting from the exclusion criteria stands out as a limitation of the study, as it possibly excluded older people with the two conditions addressed in the study. Another limitation is the absence of a sample calculation, which allows the occurrence of methodological errors, such as type II error (beta).

## CONCLUSION

Poor sleep quality was not associated with the risk of dysphagia in older adults admitted to ICUs. However, choking and respiratory incoordination were signs present in the study population. Future research is needed to verify these parameters in a larger population and better validate the findings.
